# PPAR**γ** Expression and Function in Mycobacterial Infection: Roles in Lipid Metabolism, Immunity, and Bacterial Killing

**DOI:** 10.1155/2012/383829

**Published:** 2012-07-17

**Authors:** Patricia E. Almeida, Alan Brito Carneiro, Adriana R. Silva, Patricia T. Bozza

**Affiliations:** ^1^Laboratório de Imunofarmacologia, Instituto Oswaldo Cruz, Fundação Oswaldo Cruz, 21045-900 Rio de Janeiro, RJ, Brazil; ^2^Laboratório de Biologia Celular, Departamento de Biologia, Universidade Federal de Juiz de Fora, 36036-900 Juiz de Fora, MG, Brazil

## Abstract

Tuberculosis continues to be a global health threat, with drug resistance and HIV coinfection presenting challenges for its control. *Mycobacterium tuberculosis*, the etiological agent of tuberculosis, is a highly adapted pathogen that has evolved different strategies to subvert the immune and metabolic responses of host cells. Although the significance of peroxisome proliferator-activated receptor gamma (PPAR*γ*) activation by mycobacteria is not fully understood, recent findings are beginning to uncover a critical role for PPAR*γ* during mycobacterial infection. Here, we will review the molecular mechanisms that regulate PPAR*γ* expression and function during mycobacterial infection. Current evidence indicates that mycobacterial infection causes a time-dependent increase in PPAR*γ* expression through mechanisms that involve pattern recognition receptor activation. Mycobacterial triggered increased PPAR*γ* expression and activation lead to increased lipid droplet formation and downmodulation of macrophage response, suggesting that PPAR*γ* expression might aid the mycobacteria in circumventing the host response acting as an escape mechanism. Indeed, inhibition of PPAR*γ* enhances mycobacterial killing capacity of macrophages, suggesting a role of PPAR*γ* in favoring the establishment of chronic infection. Collectively, PPAR*γ* is emerging as a regulator of tuberculosis pathogenesis and an attractive target for the development of adjunctive tuberculosis therapies.

## 1. Introduction

Tuberculosis is a global public health problem, with over 9 million new cases being reported each year that are responsible for almost 2 million deaths annually worldwide [1]. *Mycobacterium  tuberculosis* (*M. tuberculosis*), the etiological agent of tuberculosis, is a highly successful pathogen, infecting approximately one-third of the human population, and it has adapted to live within the hostile macrophage environment. Through long-standing coevolution with its mammalian host, *M. tuberculosis *has evolved different strategies to subvert the immune and metabolic responses of the host cells. Pathogenic species of mycobacteria express and regulate numerous genes within the host to evade the host immune responses and suit their intracellular life style. Among the intracellularly induced genes, several genes have functions in lipid metabolism. 

PPAR*γ* is a member of the lipid-activated nuclear receptor superfamily and plays a recognized role in the transcriptional regulation of cellular proliferation, differentiation, and inflammation in addition to metabolic regulation of lipids and glucose [2, 3]. This receptor is regulated by fatty acid metabolites and acts as a transcription factor, forming heterodimers with the retinoid X receptor (RXR) and binding to specific PPAR response elements (PPREs) in the promoter regions of target genes [4, 5]. PPARs were originally described in adipocytes, monocytes, and macrophages [6, 7]. Since then, they have been described in other immune cell types of hematopoietic origin, including T lymphocytes, B lymphocytes, NK cells, dendritic cells, neutrophils, eosinophils, and mast cells, where a role for these receptors in inflammation and immunoregulation has been proposed [7–10]. However, the role of PPARs in the host immune responses to intracellular infectious agents is only now being recognized.

Herein, we focus on the role of PPAR*γ* in intracellular bacterial infection. Specifically, we discuss the host response to *Mycobacterium* infection related to the regulation of PPAR*γ* expression by mycobacteria and PPAR*γ*-dependent effects on mycobacterial-induced modulation of host cell lipid metabolism and immune responses. Notably, PPAR*γ* expression is highly upregulated during mycobacterial infection. Mycobacterial-induced PPAR*γ* plays roles in host cell metabolism leading to increased lipid droplet formation and downregulates the host immune response to favor pathogen burden, thereby suggesting that pathogens may stimulate PPAR*γ* activity as an escape mechanism.

## 2. * Mycobacterium * Infection Triggers Increased**** PPAR**** *γ* **** Expression

PPAR*γ* is widely expressed in many cell types in different tissues, including in macrophages and dendritic cells in the lung [2, 11, 12]. Moreover, cytokines and pathogen-derived components may regulate PPAR*γ* expression in cells of the immune system [13]. Recent studies have demonstrated that mycobacterial infection significantly increases PPAR*γ* expression in human and mouse macrophages with important consequences for immune and metabolic host responses to infection [14, 15]. 

Infection of macrophages with either *M. bovis* bacillus Calmette-Guérin (BCG) or *M. tuberculosis* triggers a time-dependent increase in the expression of PPAR in macrophages *in vitro* [14, 15] and *in viv*o in the lung [16]. Increased PPAR*γ* expression was apparent as early as 2 h after infection and reached maximal levels within 24 h after the infection. Of note, non-pathogenic, fast-growing *M. smegmatis* fails to induce PPAR*γ* expression in macrophages, suggesting that PPAR*γ* expression may be related to bacterial pathogenesis [14, 15]. 

The mechanisms involved in mycobacterial-induced PPAR*γ* expression have recently been investigated. Interestingly, even infection with dead bacteria triggers PPAR*γ* expression, as paraformaldehyde-killed *M. tuberculosis* or cell-wall components; mostly mannose-caped lipoarabinomannan (ManLAM) from either BCG or *M. tuberculosis*, are able to induce PPAR*γ* expression, suggesting the role of pattern recognition receptors in the regulation of PPAR*γ* [14–16]. 

During the infection of foam-like-macrophages, pathogenic mycobacteria trigger an innate immune response mediated by pathogen-associated molecular patterns (PAMPs), such as Toll-like receptors (TLR) and NOD-like receptors (NLRs). Recent reports indicate that NLR and TLR pathways are nonredundant in the recognition of *M. tuberculosis *and can synergize to induce a proinflammatory response [17].

TLRs represent some of the most important pattern recognition receptors (PRRs) that recognize mycobacterial products [18, 19]. Recognition through TLRs results in the rapid activation of signal-dependent transcription factors, including members of the nuclear factor-*κ*B (NF-*κ*B), activator protein 1 (AP1), and interferon regulatory factor (IRF) families [20, 21]. Activation of multiple TLRs, including TLR2, TLR4, and TLR9, as well as TLR6 and TLR1 when dimerized with TLR2, contributes to an efficient innate response against mycobacterial infection, resulting in inflammatory responses with cytokine production [18, 19, 22–24]. The NOD proteins are localized in the cytoplasm, and NOD2 has been implicated in the recognition of intracellular pathogens, such as mycobacteria [17, 25, 26]. NOD2 does not play a significant role in controlling *M. tuberculosis* growth during early infection [27], although NOD2 mRNA levels are increased in patients with tuberculosis [28]. In contrast, Brooks et al. [29] reported that NOD2 controls the growth of both *M. tuberculosis* and BCG in human macrophages, whereas it controls only BCG growth in murine macrophages. Collectively, these findings suggest that activation of different pathways is important and leads to different outcomes during mycobacterial infection.

The role of TLR in regulating PPAR*γ* expression has been investigated. We demonstrated that PPAR*γ* expression in macrophages infected with BCG or stimulated with ManLAM is requisitely dependent on TLR2 signaling [14]. However, the nonpathogenic *M*. *smegmatis*, a well-known TLR2 ligand, and the synthetic TLR2 ligand Pam3Cys fail to induce PPAR*γ* expression in macrophages [14, 15], suggesting that coreceptors of TLR2 are required to induce PPAR*γ* expression. 

The TLR2 coreceptors and the downstream pathways involved in mycobacteria-induced PPAR*γ* expression are currently unknown. Of note, Rajaram et al. [15] demonstrated that infection with virulent *M. tuberculosis *or the addition of ManLAM upregulates PPAR*γ* expression independent of NF-*κ*B in human macrophages. 

## 3. PPAR*γ* Regulates Host Immune Responses to**** Mycobacterial Infection 

The host immune response to mycobacterial infection requires tightly balanced orchestration of both innate and adaptive immunity. The role of PPAR*γ* in regulating the immune responses of murine and human macrophages to different species of *Mycobacterium* has been studied. PPAR*γ* activation was demonstrated during infection by BCG [14, 16] and* M. tuberculosis* [15], as well as its major cell-wall immune-regulatory lipoglycan, namely, ManLAM [14, 15] that culminates with an anti-inflammatory response and downregulation of macrophage functions. 

Of major interest during pathogen infection, PPAR*γ* may repress target inflammatory genes, including proinflammatory cytokines and inducible NO synthase (iNOS) [30–32]. The molecular mechanisms of the negative regulation of inflammatory responses are executed, at least in part, by the ability of PPAR*γ* to interfere with the activities of other signal-dependent transcription factors by transrepression [11]. PPAR*γ*, which binds constitutively to DNA as a heterodimer with RXRs, functions as a transcriptional repressor through ligand-dependent transrepression of NF-*κ*B target genes and may also function in the absence of ligand by interacting with corepressor complexes containing histone deacetylases (HDACs), nuclear-receptor corepressor (NcoR), or the silencing mediator of retinoic acid and thyroid-hormone receptor (SMRT) [30, 31, 33]. These protein complexes bind to the promoters of inflammatory genes and prevent the acetylation of histones and the aggregation of coactivator complexes. PPAR*γ* downregulates proinflammatory gene expression by antagonizing the activity of transcription factors, including FOXP3, T-bet, and GATA-3, which are involved, respectively, in the regulation of inflammation and Th1 and Th2 immune responses [2, 3]. PPAR*γ* serves also as a negative regulator of macrophage activation, altering the expression of many inflammatory genes [7, 9], modulating macrophage differentiation and activation through transrepression of the transcription factors STAT, AP-1, and NF-*κ*B [32], and attenuating the respiratory burst [34]. The PPAR*γ* ligands induce an allosteric change in PPAR*γ* that results in covalent attachment of small ubiquitin-related modifier 1 (SUMO1) to the ligand-biding domain of PPAR*γ* using the ubiquitin-conjugating enzyme 9 (UBC9) and the protein inhibitor of activated STAT1 (PIAS1) as the SUMO E2 and E3 ligases, respectively, for transcriptional repression [11]. Next, following sumoylation, PPAR*γ* interacts with the nuclear corepressor (NCoR) complex to prevent signal-dependent recruitment of ubiquitin-conjugating enzymes (such as UBCH5) and the 19S proteasome components necessary for NCoR clearance [31]. As a result, the NCoR complex remains bound to the promoter region and exerts repressive activity to the nuclear transcription factors. 

The function of PPAR*γ* activation in the immune response to mycobacterial infection was investigated. PPAR*γ* was shown to positively regulate prostaglandin (PG) E_2_ production in BCG infected macrophages [14], a process potentiated by PPAR*γ* agonists and inhibited by antagonists. Accordingly, PPAR*γ* activation led to increased cyclooxygenase (COX) 2 expression [15] and PGE_2_ production [35] in *M. tuberculosis* infected macrophages. Of note, PGE_2_ is a potent immune modulator that downregulates Th1 responses and bactericidal activity toward intracellular organisms [36, 37]. 

The production of nitric oxide (NO) and other reactive nitrogen intermediates by innate immune cells is considered an effective host-defense mechanism against microbial pathogens, including mycobacterial infection. During infection, NO is produced by inducible NO synthase (iNOS) in response to bacterial components or a combination of proinflammatory cytokines, such as interferon (IFN)-*γ*, TNF-*α*, and IL-1*β* [38]. In most cells, iNOS transcription requires activation of NF-*κ*B by TNF-*α* and IL-1*β* and activation of STAT-1 by IFN-*γ* [39, 40]. PPAR*γ* has been implicated in the suppression of iNOS expression in macrophages [7, 32]. Synthetic PPAR*γ* agonists promote PIAS1-dependent conjugation of SUMO1 to the PPAR*γ* ligand-binding domain, preventing the signal-dependent ubiquitylation and the clearance of the NCoR complex required for full-gene activation and preventing the expression of iNOS [31]. Production of NO in macrophages is also regulated by the levels of arginases, which compete with iNOS for the substrate L-arginine, and catalyze the hydrolysis of L-arginine to L-ornithine and urea. Of note, PPAR*γ* positively regulates arginase I expression in macrophages [41]. A role of PPAR*γ* in modulating NO production during *M. tuberculosis* infection has been demonstrated. Silencing of PPAR*γ* in *M. tuberculosis* infected macrophages significantly enhanced iNOS expression and NO production in these cells while inhibited arginase I expression, suggesting an endogenous role for PPAR*γ* in the downmodulation of NO production during infection [35]. 

Infection in susceptible hosts are modulated by type 2 immune response with Th2 cells that produce IL-4 and IL-13 while protection is associated with type 1 immune response largely dependent of TNF-*α* and IFN-*γ* [42, 43]. IL-4 has been demonstrated as a key activator of PPAR*γ* by regulating the induction of the 12/15-lipoxygenase-derived PPAR*γ* ligands and through an interaction between PPAR*γ* and signal transducer and activators of transcription 6 (STAT6) on promoters of PPAR*γ* target genes [44, 45]. PPAR*γ* activation suppresses the production of proinflammatory cytokines, and are critical for the formation, activation, and maintenance of alternatively activated macrophages [45]. Elevated PPAR*γ* expression in human macrophages is one of the biological markers of IL-4/IL-13-mediated alternative activation. Conversely, deletion of PPAR*γ* in alternatively activated macrophages leads to a Th1 pulmonary inflammatory response that favor intracellular pathogen killing [46]. PPAR*γ* expression has been shown to be elevated in human alveolar macrophages, which are characterized as alternatively activated macrophages [15]. Moreover, markers of alternative macrophages are induced in *M. tuberculosis*-infected macrophages through PPAR*γ*-dependent mechanisms [35]. In addition, the balance between the activities of NF-*κ*B p65 and PPAR*γ* has been demonstrated during mycobacterial challenge. Lagranderie et al. [16] showed that in nuclear lung-cell extracts 24 h after challenge with freeze-dried BCG, PPAR*γ* expression increased and NF-*κ*B p65 expression decreased, suggesting an association between the regulation of these two factors. Moreover, PPAR*γ* knockdown in macrophages led to enhanced TNF-**α** and decreased IL-10 production by *M. tuberculosis-*infected macrophages [15, 35], indicating that PPAR*γ* activation lead to an increase IL-10/TNF ratio creating an anti-inflammatory environment favorable for pathogen growth. Together, accumulating data on PPAR*γ*-dependent effects on immune response during mycobacterial infection suggest that PPAR*γ* induction is advantageous for this host-adapted intracellular pathogen within the lung microenvironment. 

## 4. PPAR*γ* Regulates Host Metabolism to ****Mycobacterial Infection 

PPAR*γ* has been shown to function as a key transcriptional regulator of lipid metabolism in macrophages and dendritic cells (DC) (for review, see [3]) through the direct regulation of genes participating in lipid uptake, transport, and storage [47–49]. Indeed, PPAR*γ* is robustly expressed in macrophage-derived foam cells within atherosclerotic lesions, where it plays an important role in lipid homeostasis and metabolism [8, 32, 47, 50]. 

Pathogen-triggered dysregulation of host-cell lipid metabolism is emerging as a key feature in the pathogenesis of mycobacterial infection, as mycobacteria relies largely on host lipids for their survival and growth. Accumulating evidence suggests that modulation of host lipid metabolism through mycobacteria-induced lipid droplet formation is important in tuberculosis and leprosy. Foamy-like macrophages have been shown to play important roles in tuberculosis pathogenesis, both within the initial phases of macrophage infection and in granulomas [37, 51, 52]. In addition, lipid droplets formed in response to BCG and *M. leprae* constitute sites for eicosanoid synthesis, ultimately leading to increased production of PGE_2_ by infected macrophages [14, 37, 53]. 

PPAR*γ* is regulated and active in lipid droplet-enriched cells, and PPAR*γ* may regulate processes associated with lipid-droplet formation in leukocytes during intracellular mycobacterial infection. In agreement with these results, the PPAR*γ* agonist BRL49653 potentiates lipid droplet formation and PGE_2_ production induced by a suboptimal dose of BCG. Conversely, pretreatment with an antagonist of PPAR*γ* (GW9662) significantly inhibits BCG-induced lipid droplet formation and PGE_2_ production [14], indicating the requirement for PPAR*γ* signaling in lipid droplet biogenesis and further prostanoid production during BCG infection. The role of PPAR*γ* activation in regulating lipid droplet biogenesis and PGE_2_ production was subsequently confirmed in *M. tuberculosis* infected macrophages after PPAR*γ* knockdown by RNAi [35].

The mechanisms involved in PPAR*γ*-induced lipid droplet biogenesis in mycobacterial infection are still not completely understood. PPAR*γ*-mediated expression of adipose differentiation-related protein (ADRP) has been described in different cells and conditions [54, 55]. ADRP is a member of the PAT family of proteins that plays an important role in adipocyte differentiation, lipolysis modulation, lipid droplet assembly, and biogenesis (reviewed in [56]). ADRP may act as a nucleation center for the assembly of nascent lipids [57, 58] and is also associated with the surface of lipid droplets in macrophages and Schwann cells during mycobacterial infection [37, 59], which is thought to play a major role in the maintenance of lipid storage and survival of pathogens. Increased expression of scavenger receptors, including MARCO, macrophage scavenger receptor (MRS), and CD36, has been observed in mycobacterial infection and leads to increased uptake and accumulation of host-derived oxidized lipids in infected cells [60]. Conversely, enhancing cholesterol efflux by liver X receptor (LXR) activation with the synthetic agonist GW3965 significantly decreased the cholesterol ester content of cells triggered by TLR pathways, including exposure to *C. pneumonia* and LPS [61]. In addition, treatment with the fatty acid synthase inhibitor C75, a PPAR*γ* target, has been shown to inhibit significantly lipid droplet formation induced by mycobacterial infection with or without apoptotic cells, confirming the role of new lipid synthesis in lipid droplet biogenesis [62]. Thus, accumulating evidence indicates that mechanisms of increased lipogenesis, decreased lipid degradation, and regulation of lipid influx/efflux act synergistically to form lipid droplets during infection. Based on the different targets of PPAR*γ* in lipid metabolism, it is conceivable that PPAR*γ* operates at different levels to regulate lipid droplet biogenesis during infection.

## 5. Are There Roles for PPAR*γ* in Mycobacterial**** Killing and Escape Mechanisms? 

PPAR*γ* has been extensively investigated for its role in many inflammatory diseases; however, its immunoregulatory roles in infectious and parasitic diseases have only recently gained recognition (review [63]).

As discussed above, increased PPAR*γ* expression during mycobacterial infection is important for lipid metabolism and inflammatory responses of macrophages. Accumulating evidence has suggested that lipid droplet formation may favor intracellular survival and/or replication of *M. tuberculosis,* BCG and *M. leprae *in different models [37, 52, 59, 64]. Moreover, decreased production of proinflammatory cytokines and NO could also contribute to a favorable environment for pathogens, thereby suggesting that mycobacterial-induced PPAR*γ* expression may act as an escape mechanism for this intracellular parasite. 

The impact of PPAR*γ* expression and activation in mycobacterial survival within macrophages has been investigated. The role of pharmacological inhibition of PPAR*γ* in macrophage-induced mycobacterial killing was investigated. Pretreatment with the selective PPAR*γ* antagonist, GW9662, significantly enhanced the capacity of macrophages to kill BCG, as determined by live/dead bacterial staining assessed by flow cytometry [14]. The role of PPAR*γ* in modulating intracellular bacterial killing was later confirmed by silencing PPAR*γ* in human macrophages and subsequently infecting the cells with *M. tuberculosis*. Following PPAR*γ* knockdown, macrophages had significantly better ability to control *M. tuberculosis* growth, as assessed by colony forming assays [15]. The increased control of mycobacterial infection was concomitant with an increase in TNF-*α* production [15] and a decreased formation of lipid droplets [14], providing evidence that mycobacterial-induced PPAR*γ* is an important mechanism in favoring mycobacterial growth in macrophages, at least partly through transcriptional regulation of inflammatory cytokines and lipid metabolism. This finding also suggests that lipid droplets may play a role in the pathogenesis of mycobacterial infection via PPAR*γ* expression and activation dependent mechanisms.

Collectively, these findings indicate that mycobacteria utilize PPAR*γ* signaling as an escape mechanism that enables survival within the hostile environments of macrophages. 

## 6. Concluding Remarks and Perspectives

**Figure 1:**
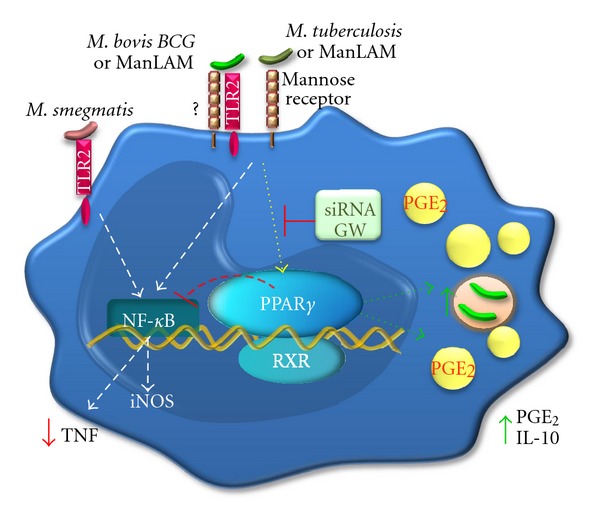
Model of PPAR*γ* functions in mycobacterial infection. The activation of macrophage TLR2 signaling by *M. tuberculosis*, ManLAM, or *M. bovis* BCG results in the activation of PPAR*γ* and NF-*κ*B. The activation of PPAR*γ* by mycobacterial infection induces lipid droplet formation, PGE_2_ production and favors mycobacterial survival. Moreover, the PPAR*γ* activation can down-modulate NF-*κ*B activity inhibiting the proinflammatory cytokine production. Both inhibition of PPAR*γ* with the selective antagonist GW9662 or PPAR*γ* knockdown with siRNA result in a reduction of lipid droplet biogenesis and increased *Mycobacterium* killing by macrophages.

Recent studies have begun to shed light on the roles of PPAR*γ* in mycobacterial infection. Studies on PPAR*γ* expression and function have revealed that this transcription factor is highly upregulated during intracellular pathogen infection in which PPAR*γ* plays roles in host cell metabolism and downregulating the host immune response to favor pathogen burden, thereby suggesting that pathogens may stimulate PPAR*γ* activity as an escape mechanism (Figure 1). Accordingly, inhibition of PPAR*γ* activity leads to increased mycobacterial killing and infection control, and as such, PPAR*γ* is emerging as an attractive target candidate for therapeutic intervention strategies.

Although great advances in the understanding of the mechanisms of pathogen-induced PPAR*γ* expression and its roles in lipid metabolism and inflammatory mediator production have been achieved, critical questions on intracellular pathogen infection remain. Future studies in animal models, as well as clinical studies, will be necessary to characterize the role of PPAR*γ* in the pathogenesis of tuberculosis and as a target for therapeutic intervention.
